# Diabetes and gender incongruence: frequent mental health issues but comparable metabolic control – a DPV registry study

**DOI:** 10.3389/fendo.2023.1240104

**Published:** 2024-01-22

**Authors:** Claudia Boettcher, Sascha R. Tittel, Felix Reschke, Maria Fritsch, Felix Schreiner, Maike Achenbach, Susanne Thiele-Schmitz, Anton Gillessen, Angela Galler, Nicole Nellen-Hellmuth, Sven Golembowski, Reinhard W. Holl

**Affiliations:** ^1^ Paediatric Endocrinology and Diabetology, University of Bern Faculty of Medicine, Bern, Switzerland; ^2^ Institute of Epidemiology and Medical Biometry (ZIBMT), University of Ulm, Ulm, Germany; ^3^ German Centre for Diabetes Research (DZD), Munich-Neuherberg, Germany; ^4^ Diabetes Centre for Children and Adolescents, Kinder- und Jugendkrankenhaus Auf der Bult, Hannover, Germany; ^5^ Department of Paediatric and Adolescent Medicine, Division of General Paediatrics, Medical University of Graz, Graz, Austria; ^6^ Paediatric Endocrinology Division, Children’s Hospital, University of Bonn, Bonn, Germany; ^7^ Department of Gastroenterology and Diabetology, Vivantes Klinikum Kaulsdorf, Berlin, Germany; ^8^ Diabetes Centre for Children and Adolescents, St. Louise Women’s and Children’s Hospital, Paderborn, Germany; ^9^ Department of Internal Medicine, Herz-Jesu-Hospital, Muenster, Germany; ^10^ Charité - Universitätsmedizin Berlin, corporate member of Freie Universität Berlin und Humboldt-Universität zu Berlin, Sozialpädiatrisches Zentrum, Paediatric Endocrinology and Diabetology, Berlin, Germany; ^11^ Centre of Child and Adolescent Medicine, Leopoldina Clinic Schweinfurt, Schweinfurt, Germany; ^12^ Department of Paediatrics, Sana Hospital Lichtenberg, Berlin, Germany

**Keywords:** diabetes mellitus, type 1 and 2, gender incongruence, metabolic control, mental health

## Abstract

**Context:**

The condition when a person’s gender identity does not match the sex assigned at birth is called gender incongruence (GI). Numbers of GI people seeking medical care increased tremendously over the last decade. Diabetes mellitus is a severe and lifelong disease. GI combined with diabetes may potentiate into a burdensome package for affected people.

**Objective:**

The study aimed to characterize people with GI and diabetes from an extensive standardized registry, the Prospective Diabetes Follow-up Registry (DPV), and to identify potential metabolic and psychological burdens.

**Methods:**

We compared demographic and clinical registry data of persons with type 1 or type 2 diabetes and GI to those without GI and used propensity score matching (1:4) with age, diabetes duration and treatment year as covariates.

**Results:**

75 persons with GI, 49 with type 1 and 26 with type 2 diabetes were identified. HbA1c values were similar in matched persons with type 1 or 2 diabetes and GI compared to those without GI. Lipid profiles showed no difference, neither in type 1 nor in type 2 diabetes. Diastolic blood pressure was higher in the type 1 and GI group than in those without, whereas systolic blood pressure showed comparable results in all groups. Depression and anxiety were significantly higher in GI people (type 1 and 2). Non-suicidal self-injurious behaviour was more common in type 1 and GI, as was suicidality in type 2 with GI.

**Conclusion:**

Mental health issues are frequent in people with diabetes and GI and need to be specially addressed in this population.

## Introduction

Gender incongruence (GI) means a person’s gender identity does not align with the sex assigned at birth. In recent years, people with GI have become more visible, and the number of people with GI seeking professional help is growing: The Amsterdam gender identity clinic saw a 20fold increase over 35 years in the number of people assessed (1). A population-based Swedish study in 50’157 adults counted 2.8% of participants who reported the desire to live or be treated as a person of another sex (2). Across children and adolescents, self-reported GI ranges between 1.3 and 2.7% in interview-based surveys [reviewed by Goodmann et al. (3)].

People with GI are at high risk for mental health issues (4): An Australian Survey found a lifetime diagnosis of depression in GI adults as high as 73% (5). For GI youth and young adults Newcomb et al. (6) reported a depression rate of more than 55%.

Diabetes mellitus – type 1 and type 2 – is a demanding illness despite the availability of effective treatments that places a significant self-management burden on affected individuals and families (7). A few years ago, the question arose whether type 1 diabetes might be more prevalent in people with GI (or vice versa) than in the general population (8–10). Another hypothesis postulated a possible link between type 2 diabetes and hormonal treatment in GI people (11). Aware that clear answers may be impossible, those questions nevertheless stimulated us to characterize the group of people with GI in an extensive international diabetes database, the DPV-registry (Diabetes prospective follow-up). We aimed to identify potential metabolic and psychological burdens in this vulnerable population.

## Materials and methods

### Study population and data source

**Figure 1:**
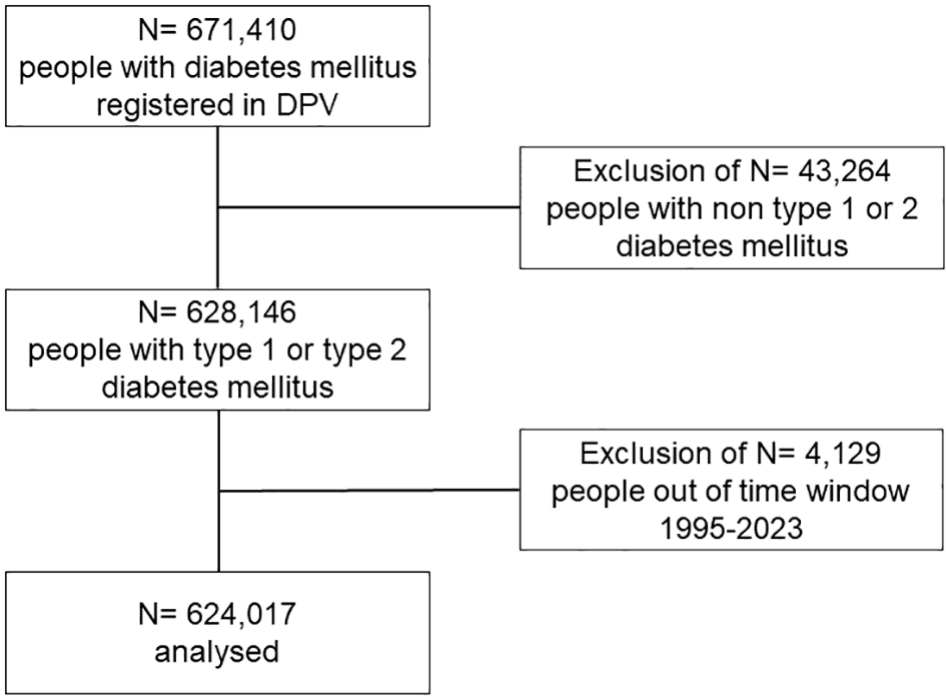
Flowchart selection of study population.

Our cohort study compared people with type 1 or 2 diabetes and concomitant gender incongruence (GI) to those without GI. Patients included in the study were extracted from the DPV registry that supports quality assurance and scientific diabetes research over all age groups. The 502 participating centres are located in Germany, Austria, Switzerland and Luxembourg. In the DPV database, 671,410 people with all types of diabetes are registered, thereof 624,017 with type 1 or type 2 diabetes between the years 1995 and June 2023 (**Figure 1**). Analysis of anonymized DPV data has ethical approval by the institutional review board of the University of Ulm, Ulm (314/21), and data collection by local review boards.

### Variables

Demographic data of our study group included age, type and duration of diabetes, sex, and gender identity. Clinical data were height, weight, calculated BMI, and BMI-SDS (12) in persons ≤ 18 years, systolic and diastolic blood pressure, and antidiabetic treatment (lifestyle intervention, oral antidiabetic agents/GLP1-agonists, insulin, or combinations). The modality of insulin treatment in type 1 diabetes was categorised as either insulin pump- or injection therapy. Continuous glucose monitoring (CGM) or flash glucose monitoring was documented. HbA1c levels reflected glycaemic control. In order to normalise for different laboratory methods, HbA1c data were mathematically standardised to the Diabetes Control and Complications Trial reference range (4.05–6.05% [21–43mmol/mol]) (13). Parameters for fat metabolism included total cholesterol, HDL-cholesterol, LDL-cholesterol and triglycerides. Data from the most recent year of follow-up were analysed for each individual. GI was assumed if ICD10 F64 or appropriate terms (e.g. transgender, trans-sexualism, gender dysphoria, gender non-conformity) in so-called free text fields of the documentation software appeared as a diagnosis. Free text fields allow data documentation without and beyond formalized ICD codes and procedures. Information about gender identity, gender-affirming surgery and hormonal treatment came from the DPVs master data sets (tick in the field transgender male or female) and free text fields. Depression, anxiety or obsessive-compulsive disorder, non-suicidal self-injury and suicidality were also assigned via documented ICD10 codes and free text fields. The number of recorded cigarettes per day led to the categorisation into smokers (≥1 cigarette/day) vs non-smokers.

### Statistical analyses

Descriptive analysis included median and quartiles for continuous variables and percentages for categorical variables. We used Wilcoxon’s rank sum test and the Chi-Square test for unadjusted comparisons. Propensity score matching with age, diabetes duration, and treatment year as covariates ensured that people with a combination of diabetes and GI and a respective matched cohort of people with diabetes only had similar baseline characteristics. Matching was conducted with a 1:4 matching process (greedy matching algorithm). The lowest possible ratio was used to minimise bias while also keeping the standardised differences of matching variables <10%. Calculated standardised differences evaluated the balancing of covariates between people with and without GI. A standardised difference of less than 10% reveals a negligible imbalance. All analyses were performed using SAS for Windows, version 9.4 (TS1M7), on a windows server mainframe.

## Results

The study population before matching comprised 162,356 people with type 1 diabetes and 461,586 people with type 2 diabetes; seventy-five persons had a documented gender incongruence, 49 with type 1 diabetes and 26 with type 2 diabetes. In the propensity-score-matched cohort, we matched the persons with type 1 diabetes and GI with 196 people without GI and those with type 2 diabetes and GI with 104 without GI. In this matched cohort, the standardized differences for the variables duration of diabetes, age and treatment year were 5.2%, 0.2% and 0.5% in the type 1 group and 9.3%, 0.4% and 9.3% in the type 2 group, demonstrating only minor differences. **Table 1** shows the characteristics of the population, unmatched and matched.

**Table 1:** Characteristics of the study population categorised into people with type 1 and 2 diabetes, with or without gender incongruence, including propensity score matched cohort without gender incongruence.

	Type 1 diabetes						Type 2 diabetes					
	No Gender Incongruence unmatched		Gender Incongruence		No Gender Incongruence matched		No Gender Incongruence unmatched		Gender Incongruence		No Gender Incongruence matched	
Parameter	n	%	n	%	n	%	n	%	n	%	n	%
**Sex female/male (%) as documented**	162,356	47/53***	49	70/30	196	53/47	461,586	47/53	26	65/35	104	41/57
	n	Median (IQR)	n	Median (IQR)	n	Median (IQR)	n	Median (IQR)	n	Median (IQR)	n	Median (IQR)
**Age (years)**	162,356	18.0 (15.0-38.9)	49	17.4 (15.2-19.5)	196	16.2 (11.3-19.33)	461,586	70.3*** (60.1-78.5)	26	48.7 (36.3-57.1)	104	45.4 (26.6-59.6)
**Age at diabetes manifestation**	162,356	11.7* (6.9-20.7)	49	8.9 (6.4-11.6)	196	9.2 (6.0-12.6)	461,586	58.6*** (48.7-68.2)	26	37.4 (27.2-53.3)	104	39.7 (25.4-51.2)
**Diabetes duration**	162,356	7.3 (2.8-14.2)	49	8.4 (5.6-12.2)	196	7.4 (2.2-12.8)	461,586	8.7* (2.9-15.6)	26	2.8 (1.2-9.8)	104	2.3 (0.2-6.8)
**Treatment year**	162,356	2016*** (2010-2022)	49	2022 (2020-2022)	196	2022 (2020-2022)	461,586	2014** (2009-2018)	26	2019 (2014-2020)	104	2018 (2013-2021)
**BMI (kg/m^2^)**	152,684	22.9 (20.0-26.2)	47	23.0 (21.2-26.6)	192	21.4 (18.4-25.3)	405,210	29.7*** (26.1-34.3)	23	41.1 (32.4-45.6)	95	33.4 (29.0-37.5)
**BMI-SDS** **(<18 years)**	81,977	0.34* (-0.30-0.96)	33	0.89 (0.19-1.25)	139	0.18 (-0.50-1.13)	–	–	–	–	–	–
**HbA1c (%)**	153,864	7.8 (6.9-9.0)	48	8.1 (7.4-9.4)	192	7.7 (7.0-8.7)	413,179	7.2 (6.3-8.5)	23	7.4 (6.5-8.4)	97	7.4 (6.2-9.5)
**HbA1c (mmol/l)**	153,864	61.5 (52.3-74.8)	48	65.2 (57.2-79.3)	192	60.4 (53.1-71.0)	413,179	54.9 (45.2-69.4)	23	57.1 (47.9-68.1)	97	57.1 (44.4-80.4)

Data are shown as median (interquartile range) or percentage. ***p<0.0001; **p<0.01, *p <0.05; unadjusted comparison between unmatched group without and with gender incongruence if asterisk-marked in column “no gender incongruence unmatched” or between the matched groups if marked in column “gender incongruence”.

In the GI group with type 1 diabetes, 33 of 49 persons counted as female for the registry; of those female registered persons, three matched with their gender identity, indicating a completed transition, 13 had a male gender identity, and one had a “diverse” gender identity; in sixteen persons registered as female, it was unclear whether the documented sex coincided with the gender identity. Thirteen persons had registered male sex: in four of those persons, the gender identity was identical to the registered sex assumedly after complete transition; another four of the persons registered as male had a female gender identity, and in another five, the gender identity was unknown. Information about gender-affirming surgery was available for 16 out of 49: Three persons had undergone surgery; for three persons, surgery was planned, while in ten, “no actual plan for surgery” was documented. We found data about any hormonal treatment (not specified) in 22 out of 49; 15 used a kind of hormonal treatment, and seven did not. Eight persons used gonadotropin blockage, seven testosterone, two oestradiol, and one progestin.

The GI group with type 2 diabetes comprised 17 persons with registered female sex, thereof nine with a documented female gender identity, one with a male gender identity and seven persons with unknown gender identity. Nine persons registered male sex, two with female and two with male gender identity, whereas five had an unknown gender identity. Data about gender affirming surgery and hormonal treatment were fragmentary and incomplete: Six people had a documented operation, and three denied any surgery, but data from 17 persons were lacking. In eight persons, hormonal therapy was reported, mostly oestradiol, with no documentation in 18 persons.

Among the 49 people with type 1 diabetes and GI, 73.5% used continuous glucose monitoring (CGM) or flash glucose monitoring (FGM), and 61.4% had an insulin pump. The matched cohort with type 1 diabetes without GI used these techniques in 65.3% and 53.5% (p>0.05, respectively).

In the matched type 2 diabetes cohort, people with (without) GI used insulin therapy with or without additional oral antidiabetics/GLP-1 agonists in 46.2% (41.4%); 23.1% (24.0%) had lifestyle interventions only; 31.8% (34.6%) were treated with oral antidiabetic agents and/or GLP1-agonists only (all p>0.05).

All groups’ systolic blood pressure parameters were in similar ranges, but not the type 1 group’s diastolic blood pressure (**Table 2**). Parameters for lipid metabolism (total cholesterol, HDL-cholesterol, LDL-cholesterol, triglycerides) did not differ in any group.

**Table 2:** Lipid metabolism parameters and blood pressure parameters in type 1 and type 2 diabetes, with and without GI in the matched cohort.

	Type 1 diabetes				Type 2 diabetes			
	Gender Incongruence		No Gender Incongruence matched		Gender Incongruence		No Gender Incongruence matched	
Parameter	n	Median (IQR)	n	Median (IQR)	n	Median (IQR)	n	Median (IQR)
**Cholesterol (mg/dl)**	36	178.4 (157.5-199.5)	150	174.5 (146.0-204.0)	15	179.0 (153.0-208.0)	57	201.1 (165.0-229.0)
**HDL-Cholesterol (mg/dl)**	35	53.0 (44.0-72.0)	141	58.4 (46.0-68.0)	14	35.8 (32.0-44.0)	53	42.0 (35.0-51.0)
**LDL-Cholesterol (mg/dl)**	35	111.0 (82.0-122.0)	141	97.0 (78.0-126.0)	13	116.0 (98.0-124.0)	55	112.0 (87.0-151.0)
**Triglycerides (mg/dl)**	37	97.4 (78.0-137.0)	144	86.4 (63.3-132.0)	13	191.0 (128.0-383.5)	57	193.0 (147.0-311.0)
**Systolic blood pressure (mmHg)**	46	123.8 (120.0-130.0)	189	120.0 (110.0-129.5)	26	135.0 (120.0-145.0)	95	130.0 (122.0-145.0)
**Systolic blood pressure SDS**	33	1.0 (0.5-1.5)	136	0.8 (0.2-1.2)	–	–	–	–
**Diastolic blood pressure (mmHg)**	46	77.5* (70.0-80.5)	189	70.0 (64.0-78.0)	26	80.0 (75.0-88.0)	94	80.0 (70.0-90.0)
**Diastolic blood pressure SDS**	33	1.3* (0.2-1.8)	136	0.3 (-0.4-1.3)	–	–	–	–

SDS-values according to KIGGS (KIGGS) for systolic and diastolic blood pressure in persons < 18 years of age. *p <0.05.

**Table 3** shows the results for the psychiatric comorbidities depression and anxiety/compulsive disorder, suicidality and smoking habits in the matched cohort: Depression and anxiety disorder were more frequent in persons with type 1 diabetes and GI. In GI people with type 2 diabetes, depression, anxiety and suicidality were significantly more frequent than in people without GI. Non-suicidal self-injury was detectable with 4.1% in the GI type 1 cohort compared to 0% in those without GI (p<0.05). In the type 2 cohort, non-suicidal self-injury stayed unreported. Smoking was more than twice as frequent in the GI groups but did not reach the significance level.

**Table 3:** Percentages of anxiety/compulsive disorders, depression, non-suicidal self-injury, suicidality and smoking habits in the matched cohorts type 1 and 2 diabetes.

	Type 1 diabetes				Type 2 diabetes			
	Gender Incongruence		No Gender Incongruence matched		Gender Incongruence		No Gender Incongruence matched	
Parameter	n	Percentage	n	Percentage	n	Percentage	n	Percentage
**Anxiety/compulsive disorder**	49	12.2***	196	1.0	23	7.7*	104	0.00
**Depression**	49	24.5***	196	3.6	23	38.5***	104	5.8
**Non-suicidal self-injury**	49	4.1*	196	0.0	23	0.00	104	0.00
**Suicidality**	49	2.0	196	0.0	23	11.5**	104	0.00
**Smoking habit**	36	13.9	102	4.9	17	47.1	58	20.7

*p <0.05; **p<0.01; ***p<0.0001.

## Discussion

Our study´s basis is the international diabetes registry DPV, which aims to improve treatment outcomes of people with diabetes through standardized documentation, objective comparison of quality indicators, and multicentre research. More than 230 of the 502 DPV diabetes centres contributed to the present analysis. As the registry’s focus is on diabetes and less on additional, not diabetes-associated diagnoses, we have to face incomplete data in the registry concerning gender incongruence. Incompleteness might apply to the GI condition and additional information about GI, e.g. hormonal treatment. Nevertheless, with 49 persons recognized as GI out of 162,356 people with type 1 diabetes and 23 GI persons out of 461,586 people with type 2 diabetes, our study shows a similar proportion of individuals who meet the criteria for transgender based on diagnostic codes as summarized in a review by Goodman et al. (3). A direct comparison of the GI rate in the countries contributing to the DPV registry is impossible as none of those countries own a population-based GI registry or other tools to estimate their country´s GI prevalence reliably. However, a recent Danish study using the unique Danish Central Person Registry in combination with the Danish National Health Registry reported a prevalence of 1/1000 of persons with a gender incongruence diagnosis and/or a legal sex change. This prevalence is much higher than the number we found in our study, assuming some underreporting in the registry (14). That the proportion of GI people we saw in the type 2 group is lower than in the type 1 group is probably due to the age difference: The type 1 group is considerably younger than the type 2 group, and the proportion of people living with GI is reported higher in younger people (15). This phenomenon is also visible within the unmatched type 2 group: people with type 2 and GI are younger than those without GI. The age difference disappears in the matched cohort, as matching for age is part of the matching process.

In our study, we can report what medical personnel documented as sex. Due to the possibility of additionally filling in “transgender male” and “transgender female”, the documented sex corresponds mainly to the assigned sex at birth, especially in those without GI. However, some uncertainty remains in people with GI, as not everyone has a documented gender identity. In at least seven persons, the documented sex corresponds to the gender identity, not the assigned sex at birth. Our database also has incomplete data about hormonal therapy and gender-affirming surgery in GI people. Therefore, we cannot conclude, e.g. about a connection between gender-affirming hormonal treatment and metabolic state. However, a direct comparison of the groups with and without GI is possible.

Due to the matching process, we lose a significant part of the data on people with diabetes, resulting in a much smaller sample size than without the matching. The process may preclude small but clinically significant differences. But by the matching process, we could overcome the noticeable differences in age, diabetes duration and treatment year between people with and without GI and therefore create comparable groups.

In the matched type 1 diabetes group, we see a comparable metabolic control measured by HbA1c-values in people with GI and those without GI and a similar rate of CGM/FGM devices- and insulin pump use. CGM/FCM, especially in combination with insulin pumps, promises optimal metabolic control through flexible insulin dose adjustment and reduced hypoglycaemia risk (16). This result is reassuring: although far from the guidelines´ goal of an HbA1c-value of < 7% (17, 18), people with GI manage their chronic disease diabetes mellitus as successfully as those who are not facing GI. Therefore, the risk for diabetes complications seems comparable to the complication risk of their diabetes peers.

People with GI show a higher BMI than those without GI in the unmatched type 2 group. A higher BMI could be due to body dissatisfaction, as we know that body dissatisfaction in GI people can lead to disordered eating (19). Another reason for the higher BMI in the GI group with type 2 can lie in gender-affirming hormonal treatment: A review by Velho et al. (20) concluded that testosterone treatment in trans men could lead to an increase in BMI. Suppakitjanusant et al. (21) saw an increase in BMI in trans women but not in trans men at the initiation of hormonal therapy. It is noticeable that the BMI difference lost significance in the matched type 2 cohort (p=0.07): The GI effect on BMI might be marginal and only gain significance with high numbers of individuals. Generally, gender-affirming hormonal treatment is associated with a likely increased risk for dyslipidaemia in testosterone-based regimens and a likely increased risk for cardiovascular disease in testosterone- and oestrogen-based treatment in the presence of additional risk factors (22). For our GI population, we saw similar levels of cholesterol parameters and similar blood pressure profiles compared to the corresponding non-GI-diabetes groups – except for the diastolic blood pressure in the type 1 group with GI. It is uncertain whether this effect is an actual GI effect: We would expect a similar effect in the type 2 group, but this is not the case.

As our study is only a snapshot, we cannot make a statement about long-term lipid and blood pressure profile courses.

Diabetes mellitus, a chronic disease, is associated with mental health issues like depression or anxiety. A review by Farooqi et al. (23) showed a prevalence of depression of 22% in type 1 diabetes and 19% in type 2 diabetes compared to those without diabetes (13% and 11%, respectively). As a diagnosis of depression or anxiety is associated with adverse treatment outcomes in type 1 and 2 diabetes (24–26), it is essential to recognize and manage these conditions. People with GI experience a high burden of mental health problems, often suffer from so-called minority stress (high levels of stress faced by members of stigmatized minority groups) and are prone to substance use (6). An Australian national study found a diagnosis of depression (any life point) in 56% of transgender people and a diagnosis of anxiety in 38% (27). Bullying is something that adolescents and young adults with GI report to have experienced in up to 85%, resulting in greater anxiety symptomology and depression (28). A recent meta-analysis investigated non-suicidal self-injury, suicidal ideation and suicidal attempts in gender non-conforming youths up to 25 years. The analysis found a mean prevalence of non-suicidal self-injury of more than 28%, a similar prevalence for suicidal ideation and a prevalence of suicidal attempts of 14.8% (29). An online study of 700 New Zealanders and Australians (self-selecting sample) aged 30-74 years revealed an incredibly high percentage of lifetime suicidal attempts of 40.1% and lifetime self-harm of 88% in transgender people compared to 27.8% and 62.0% in cisgender people (30). In our study, we saw in GI people higher proportions of anxiety or a compulsive disorder of depression diagnosis (type 1 and type 2 group) as well as non-suicidal self-injury (type 1 group) and suicidality (type 2) than in non-GI-people. However, all our rates stayed way below the rates reported in the literature when focusing on a pure transgender population. Nevertheless, the results remind us that people with GI and diabetes are more prone to mental health issues than people with diabetes alone. A review authored by Mahfouda et al. (31) concluded that in GI adults, gender-affirming cross-sex hormone interventions and surgical interventions might be associated with a substantial reduction in psychological distress. The same was true for GI adolescents, although on a much thinner data basis. Due to missing information about cross-sex hormone and surgical interventions, we are not able to compare GI people with diabetes and the mentioned treatments to those without such therapies in our population, let alone their influence on clinical outcome and glucose metabolism.

Smoking seems to be common in up to 41% of transgender youth and young adults (6). Smoking is a health risk that may further increase cardiovascular risks and, e.g. the risk for thrombosis in individuals receiving gender-affirming hormone treatment, particularly oestrogens (22, 32). In the matched type 2 cohort, 47.1% of GI people are smokers compared to “only” 20.1% of non-GI people.

Unprejudiced treatment of GI people is vital. The combination of diabetes mellitus with non-ideal metabolic control with mental health issues plus, e.g. gender-affirming hormonal treatment plus smoking and maybe a high BMI can result in a package with serious health consequences. GI people with diabetes might experience a deepening of diabetes-related psychological issues or mental health issues due to GI might be influenced by the diabetes condition. Our results can contribute to overthinking treatment routines in diabetes teams as GI people need screening for distress, special attention, counselling and the best care to shoulder their additional burden. Ideally, regular and mandatory psychologic screening and counselling would be covered by the same person with expertise in diabetes and transgender care. A 2023 review by Campos et al. supports the positive effect of psychological interventions in GI youth and adults (33).

Our study has several limitations but also strengths. The most extensive limitation is the retrospective use of a diabetes registry not designed to answer questions about GI people. Therefore, as mentioned above, we must face incomplete data when it comes to non-diabetes information. For example, we do not know reliably know whether the GI subjects have received a formal diagnosis of GI or whether GI is accompanied by gender dysphoria. Detailed information about hormonal and surgical treatment is lacking. Due to the registry´s character, we can only describe features like mental health issues in GI people and diabetes, quantify and compare those, but we cannot draw causative conclusions. On the other hand, such a registry reflects real-life conditions and mirrors the challenges we face in daily practice outside an artificial study environment. Another strength is the registry’s multicentre approach with more than 500 international diabetes centres that dissipates single centres’ biases with the possibility of creating a matching cohort. Hence the matching process is a limitation itself, as it drastically reduces the study population.

## Conclusion

In conclusion, we could demonstrate that people with GI included in an extensive diabetes registry show similar HbA1c levels and lipid profiles in type 1 and type 2 diabetes than those without GI. People with GI suffer an unfavourable high percentage from mental health issues like depression, anxiety disorders and high suicidality that need to be addressed in the clinical context.

## Data availability statement

The raw data supporting the conclusions of this article will be made available by the authors, without undue reservation.

## Ethics statement

The studies involving humans were approved by University of Ulm, Ulm (314/21). The studies were conducted in accordance with the local legislation and institutional requirements. Written informed consent for participation was not required from the participants or the participants’ legal guardians/next of kin in accordance with the national legislation and institutional requirements.

## Author contributions

CB, ST, FR and RH contributed substantially to the conception and design of the work as well as to the analysis and interpretation of data for the work. All authors are responsible for data acquisition for the work. CB drafted the manuscript. All authors contributed to the article and approved the submitted version. All authors agree to be accountable for all aspects of the work in ensuring that questions related to the accuracy or integrity of any part of the work is appropriately investigated and resolved.
