# Supplementary material for: Diabetes and gender incongruence: frequent mental health issues but comparable metabolic control – a DPV registry study
Source: Front Endocrinol (Lausanne). 2024 Jan 22;14:1240104. doi: 10.3389/fendo.2023.1240104 (PMC10841572; doi:10.3389/fendo.2023.1240104)
Supplement: Supplementary file 1 [file Table_4.docx]

Centres that contributed to the study

| Aachen - Uni-Kinderklinik RWTH | Chemnitz Kinderklinik |
| --- | --- |
| Aalen Kinderklinik | Coesfeld/Dülmen Innere Med. |
| Aidlingen Praxisgemeinschaft | Darmstadt Innere Medizin |
| Arnsberg-Hüsten Karolinenhosp. Kinderabteilung | Darmstadt Kinderklinik Prinz. Margaret |
| Aue Helios Kinderklink | Datteln Vestische Kinderklinik |
| Augsburg IV. Med. Uni-Klinik | Deggendorf Gemeinschaftspraxis |
| Augsburg Josefinum Kinderklinik | Deggendorf Medizinische Klinik II |
| Aurich Kinderklinik | Dortmund Johannes Hospital |
| Bad Aibling Internist. Praxis | Dortmund Kinderklinik |
| Bad Driburg / Bad Hermannsborn Innere | Dortmund Knappschaftskrankenhaus Innere |
| Bad Kösen Median Kinderklinik | Dortmund Medizinische Kliniken Nord |
| Bad Reichenhall Kreisklinik Innere Med. | Dortmund-West Innere |
| Bad Säckingen Hochrheinklinik Innere | Dresden Neustadt Kinderklinik |
| Basel Uni-Kinderspital beider Basel (UKBB) | Dresden Uni-Kinderklinik |
| Bautzen Oberlausitz KK | Duisburg Homberg Helios Rhein-Ruhr Kliniken GmbH |
| Bayreuth Innere Medizin | Duisburg Sana Kinderklinik |
| Berchtesgaden CJD | Duisburg St. Anna Innere Helios Rhein-Ruhr Kliniken GmbH |
| Berlin DRK-Kliniken Mitte Innere | Duisburg-St.Johannes Helios |
| Berlin DRK-Kliniken Pädiatrie | Düren-Birkesdorf Kinderklinik |
| Berlin Endokrinologikum | Düsseldorf Uni-Kinderklinik |
| Berlin Evang. Krankenhaus Königin Elisabeth | Eberswalde Klinikum Barnim Werner Forßmann - Innere |
| Berlin Klinik St. Hedwig Innere | Eckernförde Gem.-Prax |
| Berlin Lichtenberg - Kinderklinik | Erfurt Kinderklinik |
| Berlin Oskar Zieten Krankenhaus Innere | Erlangen Uni Innere Medizin |
| Berlin Schlosspark-Klinik Innere | Erlangen Uni-Kinderklinik |
| Berlin Virchow-Kinderklinik | Essen Diabetes-SPP |
| Berlin Vivantes Hellersdorf Innere | Essen Diabetes-Schwerpunktpraxis |
| Bern Inselspital Kinderklinik | Essen Elisabeth Kinderklinik |
| Bochum Universitätskinderklinik St. Josef | Essen Kinderarztpraxis |
| Bodnegg - MVZ Wollmarshöhe | Essen Uni-Kinderklinik |
| Bonn Schwerpunktpraxis | Forchheim Diabeteszentrum SPP |
| Bonn Uni-Kinderklinik | Frankfurt Diabeteszentrum Rhein-Main-Erwachsenendiabetologie (Bürgerhospital) |
| Bottrop Knappschaftskrankenhaus Innere |  |
|  | Frankfurt Diabeteszentrum Rhein-Main-pädiat. Diabetologie (Clementine-Hospital) |
| Braunschweig Kinderarztpraxis |  |
| Bremen - Mitte Innere | Frankfurt-Sachsenhausen Innere |
| Bremen Zentralkrankenhaus Kinderklinik | Frankfurt-Sachsenhausen Innere MVZ |
| Bremerhaven Kinderklinik | Freiburg Uni Innere |
| Bruchweiler Edelsteinklinik Kinder-Reha | Freiburg Uni-Kinderklinik |
| Castrop-Rauxel Evangelisches Krankenhaus | Gaissach Fachklinik der Deutschen Rentenversicherung Bayern Süd |

| Geislingen Klinik Helfenstein Innere | Kassel Klinikum Kinder- und Jugendmedizin |
| --- | --- |
| Gelnhausen Innere | Kaufbeuren Innere Medizin |
| Gelsenkirchen Kinderklinik Marienhospital | Kempten Oberallgäu Kinderklinik |
| Gießen Ev. Krankenhaus Mittelhessen | Kiel Städtische Kinderklinik |
| Gießen Uni-Kinderklinik | Kleve Innere Medizin |
| Graz Uni-Kinderklinik | Koblenz Kinderklinik Kemperhof |
| Göttingen Uni Gastroenterologie | Konstanz Innere Klinik |
| Göttingen Uni-Kinderklinik | Konstanz Kinderklinik |
| Halberstadt Innere Med. AMEOS Klinik | Krefeld Innere Klinik |
| Halle Uni-Kinderklinik | Krefeld-Uerdingen St. Josef Innere |
| Hamburg Altonaer Kinderklinik | Kreischa-Zscheckwitz Klinik Bavaria |
| Hamburg Endokrinologikum | Köln Kinderklinik Amsterdamerstrasse |
| Hamburg Kinderklinik Wilhelmstift | Köln Uni-Kinderklinik |
| Hameln Kinderklinik | Landshut Kinderklink |
| Hamm Kinderklinik | Leer Klinikum - Klinik Kinder & Jugendmedizin |
| Hanau Kinderklinik | Leipzig Uni-Kinderklinik |
| Hanau diabetol. Schwerpunktpraxis | Leverkusen Kinderklinik |
| Hannover DM-SPP | Lienz Diabetesschwerpunktpraxis für Kinder und Jugendliche |
| Hannover Kinderklinik auf der Bult | Lilienthal Diabeteszentrum |
| Haren Kinderarztpraxis | Lindlar DM-Zentrum |
| Heide Kinderklinik | Linz Krankenhaus der Barmherzigen Schwestern Kinderklinik |
| Heidelberg Uni-Kinderklinik | Ludwigsburg Kinderklinik |
| Herdecke Kinderklinik | Ludwigshafen diabetol. SPP |
| Herford Klinikum Kinder & Jugendliche | Luxembourg - Centre Hospitalier |
| Herne Evan. Krankenhaus Innere | Lübeck Uni-Kinderklinik |
| Hildesheim Bernward Krks Kinderheilkunde | Lübeck Uni-Klinik Innere Medizin |
| Hildesheim GmbH - Innere | Lüdenscheid Märkische Kliniken - Kinder & Jugendmedizin |
| Hohenmölsen Diabeteszentrum | Lünen Klinik am Park |
| Idar Oberstein Schwerpunktpraxis | Magdeburg Uni-Kinderklinik |
| Ingolstadt Klinikum Innere | Mainz Uni-Kinderklinik |
| Innsbruck Uni-Kinderklinik | Manderscheid Rathauspraxis |
| Itzehoe Kinderklinik | Mannheim Uni-Kinderklinik |
| Jena Kinderarztpraxis | Marktredwitz Innere Medizin |
| Jena Uni-Kinderklinik | Meissen Kinderklinik Elblandklinikum |
| Kamen Klinikum Westfalen Hellmig Krankenhaus | Memmingen Internistische Praxis |
| Kamen MKK - Medizinisches Kompetenzkollegium | Moers - St. Josefskrankenhaus Innere |
| Karlsburg Klinik für Diabetes & Stoffwechsel | Moers Kinderklinik |
| Karlsruhe Schwerpunktpraxis | Murnau am Staffelsee - diabetol. SPP |
| Karlsruhe Städtische Kinderklinik | Mönchengladbach Kinderklinik Rheydt Elisabethkrankenhaus |

| Mühlheim an der Ruhr Evang. Krankenhaus Med. Klin. | | Stade Kinderklinik |
| --- | --- | --- |
| München 3. Orden Kinderklinik | | Stockerau Landeskrankenhaus |
| München Diabetes-Zentrum Süd | | Stolberg Kinderklinik |
| München Praxiszentrum Saarstrasse | | Stuttgart Olgahospital Kinderklinik |
| München von Haunersche Kinderklinik | | Sylt Rehaklinik |
| München-Schwabing Kinderklinik | | Tettnang Innere Medizin |
| Münster Herz Jesu Innere | | Traunstein Kinderklinik |
| Münster Ludgerus-Kliniken GmbH | | Traunstein diabetol. Schwerpunktpraxis |
| Münster St. Franziskus Kinderklinik | | Trier Kinderklinik der Borromäerinnen |
| Neuwied Kinderklinik Elisabeth | | Trostberg Innere |
| Neuwied Marienhaus Klinikum St. Elisabeth Innere | | Tübingen Uni-Kinderklinik |
| Nürnberg Uniklinik Med. Klinik 4 | | Ulm Uni-Kinderklinik |
| Oberhausen Innere | | Viersen internist. Praxis |
| Oberhausen Kinderklinik | | Villach Kinderklinik |
| Offenbach/Main Innere Medizin | | Villingen-Schwenningen Schwarzwald Baar Klinikum Kinderklinik |
| Offenburg Kinderklinik | | Villingen-Schwenningen Schwarzwald-Baar-Klinikum Innere |
| Oldenburg Schwerpunktpraxis Pädiatrie | | Volkertshausen Gemeinschaftspraxis |
| Osnabrück Christliches Kinderhospital | | Weiden Kinderklinik |
| Paderborn St. Vincenz Kinderklinik | | Wesel Marienhospital Kinderklinik |
| Pforzheim Kinderklinik | | Wien Klinik Ottakring (Wilhelminenspital) 5. Med. Abteilung |
| Pirmasens Städtisches Krankenhaus Innere | | Wien Preyersches Kinderspital |
| Ravensburg Kinderklink St. Nikolaus | | Wien SMZ Ost Donauspital |
| Reutlingen Kinderarztpraxis | | Wien Uni Innere Med III |
| Reutlingen Kinderklinik | | Wien Uni-Kinderklinik |
| Rosenheim Innere Medizin | | Wiesbaden Helios Horst-Schmidt-Kinderkliniken |
| Rosenheim Schwerpunktpraxis | | Wilhelmshaven Kinderarztpraxis |
| Rostock Universität Innere Medizin | | Winnenden Rems-Murr Kinderklinik |
| Rotenburg/Wümme Agaplesion Diakonieklinikum Kinderabteilung | | Witten Kinderarztpraxis |
| Rottweil Gemeinschaftspraxis für Innere Medizin | | Wittenberg Kinderklinik |
| Saaldorf-Surheim Diabetespraxis | | Wittlich DSP |
| Salzburg Universitäts-Kinderklinik | | Worms Schwerpunktpraxis |
| Scheidegg Prinzregent Luitpold | | Wuppertal Universitäts-Kinderklinik |
| Schweinfurt Kinderklinik | |  |
| Schwerin Innere Medizin | |  |
| Schwerin Kinderklinik | |  |
| Siegen Kinderklinik | |  |
| Spaichingen Innere | |  |
| St. Augustin Kinderklinik | |  |
| St. Pölten Universitätsklinik Innere |  | |
